# Comparison of the Mesiodistal Angulations of Canine and Molar Teeth in Different Types of Orthodontic Malocclusions: A Retrospective Study

**DOI:** 10.3390/diagnostics13071351

**Published:** 2023-04-05

**Authors:** Orhan Cicek, Hakan Yilmaz, Busra Demir Cicek

**Affiliations:** 1 Department of Orthodontics, Faculty of Dentistry, Zonguldak Bulent Ecevit University, 67100 Zonguldak, Türkiye; 2 Private Practice, 34555 Istanbul, Türkiye; 3 Private Practice, 67600 Zonguldak, Türkiye

**Keywords:** mesiodistal axial angulation, canine, first molar, malocclusion, panoramic radiograph, orthodontics

## Abstract

(i) Objective: Changes in the mesiodistal axial angulations of teeth with orthodontic treatment have been a topic of interest in orthodontics for many years, although it has not been clarified enough yet. Therefore, this present study aimed to compare mesiodistal axial angulations of canine and first molar teeth by measuring from pre- and post-treatment panoramic radiographs in different types of orthodontic malocclusions. (ii) Materials and Methods: In the study, the mesiodistal axial angulation angles of the lower–upper canines (teeth numbered 13, 23, 33, and 43) and first molars (teeth numbered 16, 26, 36, and 46) were compared on panoramic radiographs taken pre- (T0) and post- (T1) orthodontic treatment of 353 patients: 237 female (mean age 14.74 ± 2.96) and 116 male (mean age 14.44 ± 2.50), who had not received any prior orthodontic treatment. The groups were formed according to pre-/post-treatment, gender, angle classification, skeletal classification, bilaterally first premolar extraction/non-extraction, and the use/non-use of miniscrews in the extraction cases. The mesiodistal angulations between the long axes of both the lower and upper canines and first molars and the interorbital plane were measured separately and recorded. The reliability analysis between the repeated measurements was evaluated using the intraclass correlation coefficient (ICC). For statistical analysis, a paired sample *t*-test and Wilcoxon test were used for the normally and non-normally distributed data, respectively. For the between-groups comparison, independent sample *t*-test and one-way ANOVA were used for normally distributed data, while the Mann–Whitney U and Kruskal–Wallis tests were used for non-normally distributed data. A value of *p* < 0.05 was considered statistically significant. (iii) Results: ICCs showed excellent reliability, ranging from 0.804 to 0.913 in other teeth, yet were good in tooth 43 (ICC = 0.712). Regardless of the groups, statistically significant differences were found between the T0 and T1 angulations for all teeth, except teeth 13 and 16. In all groups, the increase in the angulations of teeth 33 and 43 and the decrease in the angulations of teeth 36 and 46 (except skeletal class 3) were found to be statistically significant. The T0 and T1 angulation changes in the miniscrews in the used and non-used groups in extraction cases were similar to the differences found in all teeth, regardless of the groups. There was no significant difference between gender, skeletal classes, and angle classes in the amounts of change in the mesiodistal angulations. (iv) Conclusion: It was concluded that orthodontic treatment caused significant changes in the mesiodistal axial angulation of the canine and the first molar teeth. Furthermore, the fact that the angulations tended to increase in the lower canine teeth and decrease in the lower first molar teeth revealed the importance of tooth movement control, especially in orthodontic mechanics in the mandibula.

## 1. Introduction

Stability of the maxillofacial complex can be achieved through the equilibrium between occlusal forces and normal function, making it very important to position the teeth in the appropriate mesiodistal angulation with orthodontic treatment [1]. One of the keys to ideally positioning teeth in the three planes of space has been reported as being the proper mesiodistal axial inclination, also defined as the mesiodistal tip by Andrews [2]. Panoramic radiographs are used for this purpose as a pre- and post-treatment record, which can evaluate the ideal alignment of teeth, and occlusion [3]. Furthermore, previous studies have reported on the evaluation of root angulations, after orthodontic treatment and during retention, with panoramic radiographs for an American Board of Orthodontics certification [4,5,6].

Panoramic radiographs, which are an important part of orthodontic diagnosis and treatment planning, are not entirely excellent diagnostic records [7]. It has been reported that their poor quality and/or distorted images may be due to various factors ranging from errors in imaging technique to incorrect head positioning [8,9]. However, Hardy et al. [10] reported that panoramic radiographs guide the placement of orthodontic brackets; hence, are an important mainstay for the assessment of the mesiodistal axial inclination of teeth. Despite all its disadvantages, the use of panoramic radiography in the evaluation of mesiodistal tooth angulation is considered standard and it is the most commonly used method in orthodontics [7,11,12,13].

Orthodontic malocclusions are caused by a discrepancy between complex growth and the development of facial structures, local and environmental factors, and the lack of any dentoalveolar compensation mechanisms, which can alleviate these discrepancies [14,15]. Related to this, Tsunori et al. [16] reported that posterior teeth had a buccal inclination in patients with vertical facial growth patterns and a lingual inclination in patients with horizontal growth patterns. Badiee et al. [17] reported that the mean angle of the posterior teeth in both normal and open bites was significantly higher than the deep bite, relative to the palatal plane, and this angulation decreased with the increase in the over bite. In addition, Su et al. [18] reported that the upper and lower first molars showed different angulations at different growth periods, with more distally tipped upper first molars in adolescents, while it became mesially tipped in adults, whereas, the lower molars had more stable angulations during different developmental stages, and the molar angulations varied among different sagittal malocclusions.

In the literature, it was seen to be related to mesiodistal angulations, whereby the changes that occur with growth were investigated or studies were carried out in small sample size malocclusion groups [17,19]. In addition, very few studies have investigated the changes in mesiodistal axial angulation following orthodontic treatment [13,20]. Therefore, it was decided that a comprehensive investigation of the changes in the mesiodistal axial angulation of the canine and first molars following orthodontic treatment was necessary.

The aim of this retrospective study was to compare mesiodistal angulations of canine and first molar teeth by measuring the pre- and post-treatment panoramic radiographs for different types of orthodontic malocclusions.

## 2. Materials and Methods

In this retrospective study, pre- and post-treatment panoramic radiographs of 353 patients (237 female, mean age 14.74 ± 2.96; 116 male, mean age 14.44 ± 2.50) who were in the peak and post-peak period were used to assess the mesiodistal axial angulation of the upper and lower canines (teeth numbered 13 (for upper right canine), 23 (for upper left canine), 33 (for lower left canine), and 43 (for lower right canine)) and first molars (teeth numbered 16 (for upper right first molar), 26 (for upper left first molar), 36 (for lower left first molar), and 46 (for lower right first molar)). Panoramic radiographs of the patients were acquired at an equal standard before (T0) and after (T1) the fixed orthodontic treatment using an X-ray device (Veraview IC5 HD, J Morita Mfg. Corp., Kyoto, Japan), irradiating in the range of 60–70 kVp and 1–7.5 mA, and examined.

The panoramic radiographs reviewed in the study were obtained from the examination of the archive records for the patients who were referred to the Department of Orthodontics. The inclusion criteria were as follows: no prior orthodontic treatment, no severe skeletal anomalies (vertical, transversal, and/or sagittal), no need for fixed or removable functional treatment, completed permanent dentition, no missing or impacted teeth, no abnormal crown/root morphology, treated with fixed preadjusted orthodontic appliance using a 0.022-inch slot MBT prescription, having a 3 mm or less crowding for non-extraction cases, having a 7 mm or more crowding for extraction cases, completed fixed orthodontic treatment, and pre- and post-treatment panoramic radiographs with good image quality and high resolution. If at least one of these criteria was not provided, it was excluded from the study.

All patients were treated with a preadjusted appliance using 0.022 × 0.028-inch Mini Master series stainless steel brackets (American Orthodontics, Sheboygan, WI, USA) in a straight wire technique, according to MBT™ prescription [21]. In non-extraction cases with mild crowding, a 0.014-inch round Tanzo™ nitinol heat-activated (American Orthodontics), 0.016-inch round Tanzo™ nitinol heat-activated (American Orthodontics), 0.016-inch round stainless steel (American Orthodontics), 0.019 × 0.025-inch rectangular Tanzo™ nitinol heat-activated (American Orthodontics), and a 0.019 × 0.025-inch rectangular stainless steel (American Orthodontics) archwires were used, in accordance with the recommended archwire sequence [21]. In extraction cases, those whose right–left first premolars were extracted bilaterally were considered. The sequence of the using archwires was similar to the non-extraction cases, and 0.019 × 0.025-inch brass posted stainless steel rectangular archwire (American Orthodontics) was placed during the space closure. Before the space closure, the archwire was left passive for 1 month for the required torque changes and final leveling. Space closure with active tie-back activation was performed with a force of 200–300 g (grams) using an elastomeric module, stretched to twice its normal size [21]. Tie-backs for space closure were activated from the miniscrew head in the miniscrew used cases, and from the first molar tube in the non-used cases to the hook of the brass posted archwire. Miniscrews were placed in cases, where maximum anchorage was required, yet not for moderate. Miniscrews (1.5 mm diameter × 8 mm length, Aarhus System, American Orthodontics) were inserted into the alveolar bone between the roots of the second premolar and the first molar with a hand screwdriver, under local anesthesia [22].

Ethical approval was obtained from the Non-Interventional Clinical Research Ethics Committee of Zonguldak Bülent Ecevit University for the study (decision no: 2020/22-8 and date: 18 November 2020). In the study, the sample size calculation was not performed because the panoramic radiographs for all the patients who met the inclusion criteria, and whose treatment was completed in the Department of Orthodontics at Zonguldak Bülent Ecevit University between 2017 and 2021 were obtained from the archive [23].

In the study, groups were comprised according to gender, angle classification, extraction and non-extraction plans, and skeletal classification. Moreover, extraction cases were divided into two groups: miniscrew used and miniscrew not used. In total there were 195 non-extraction cases, with 79 cases in skeletal class 1, skeletal class 2 included 64 cases, and skeletal class 3 included 52 cases, while there were 73 cases in angle class 1, angle class 2 included 88 cases, and angle class 3 malocclusions included 34 cases. On the other hand, there were a total of 158 extraction cases, with 80 cases in skeletal class 1, 59 cases in skeletal class 2, and 19 cases in skeletal class 3, while there were 40 cases in angle class 1, angle class 2 included 91 cases, and angle class 3 malocclusions included 27 cases. Descriptive data on the patients and malocclusions are given in Table 1 and Table 2, respectively.

In the angle classifications, those who had the same sagittal molar relationship bilaterally were considered. Groups according to angle classification were divided according to the sagittal occlusal relationships of the lower and upper first molars, as follows [24]:

- Angle class 1: normal sagittal relationship.
- Angle class 2: the lower molar is positioned more distally than the upper molar.
- Angle class 3: the lower molar is positioned more mesially than the upper molar.

Groups according to the skeletal classification were divided as follows, and considered Steiner’s ANB (the angle between the A point–Nasion-B point) angle [25]:

- Skeletal class 1: ANB angle between 0° to 4°.
- Skeletal class 2: ANB angle more than 4°.
- Skeletal class 3: ANB angle less than 0°.

**Figure 1:**
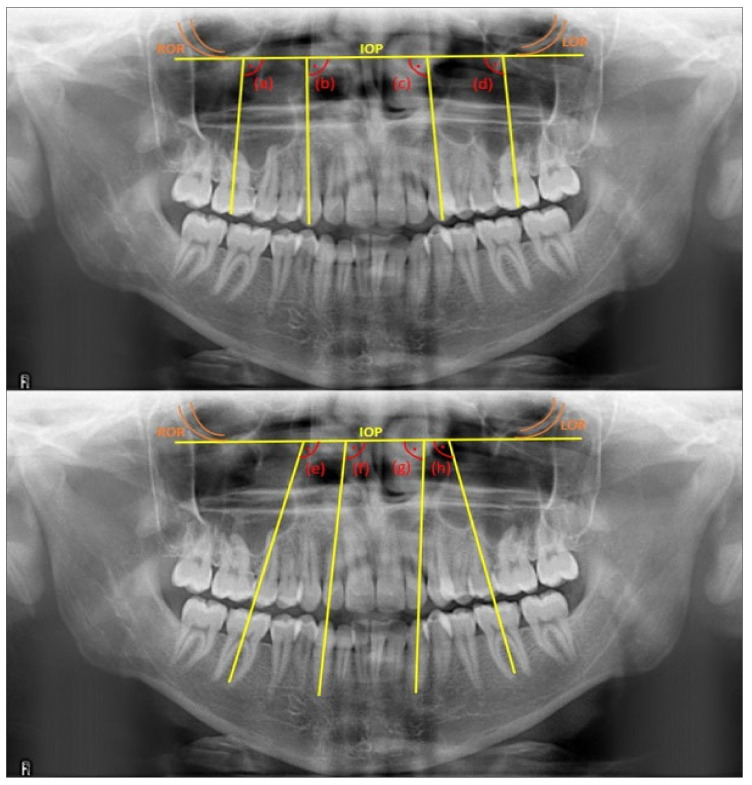
Mesiodistal axial angulation angles measured in the study: (a) for tooth 16, (b) for tooth 13, (c) for tooth 23, (d) for tooth 26, (e) for tooth 46, (f) for tooth 43, (g) for tooth 33, and (h) for tooth 36. ROR: right orbital rim; LOR: left orbital rim; IOP: interorbital plane.

The measurements in panoramic radiographs were performed using the IC Measure program (for Windows, version 2.0.0.286, The Imaging Source, LLC, Charlotte, NC, USA). The interorbital plane, which extended between the most inferior points of the right and left orbital rims, was constructed as a reference [26]. Mesiodistal axial angulation angles were measured for canines as follows: the angle between the interorbital plane and the line parallel to the long axis, combining the incisal edge and root apex, while for the first molars, it was the angle between the interorbital plane and the parallel line to the long axis, which divides the crown into two equal parts as mesiodistal [26] (see Figure 1). Measurements were performed blindly by the researcher (O.C.), according to the sequence identification number given to each sample, regardless of the groups [3].

### Statistical Analysis

Statistical analysis of the data was performed using the IBM SPSS program (version 26, SPSS, IBM Corporation, New York, NY, USA). Pre- and post-treatment means and standard deviations were calculated for each angular variable. Whether the data were normally distributed or not was evaluated with the Kolmogorov–Smirnov test. Accordingly, a paired sample *t*-test was used for the parametric evaluation of the intragroup and the Wilcoxon test was used for the non-parametric evaluations. Independent sample *t*-test and one-way ANOVA were used for the parametric evaluation of the between-groups and the Mann–Whitney U and Kruskal–Wallis tests were used for the non-parametric evaluations. The reliability test between measurements was evaluated by Cronbach’s α and two-way random effect intraclass correlation coefficients (ICCs). Probabilities less than 5% were considered statistically significant (*p* < 0.05).

## 3. Results

The differences between the repeated measurements, performed on 20 randomly selected samples after 4 weeks, were evaluated by the ICCs. The ICCs were calculated as follows: 0.912 for the upper right canine, 0.866 for the upper left canine, 0.908 for the upper right first molar, 0.878 for the upper left first molar, 0.913 for the lower left canine, 0.712 for the lower right canine, 0.804 for the lower left first molar, and 0.866 for the lower right first molar. These data showed excellent intraobserver reliability in all teeth, although only good for the lower right canine.

The angulation angles of teeth 13 and 16 did not differ significantly between T0 and T1, although there was a statistically significant decrease in the angulation angles of teeth 23 and 26 in T1 compared to T0. In T1 compared to T0, there was an increase in the angulation angles of teeth 33 and 43 and a decrease in the angulation angles of teeth 36 and 46, which were statistically significant (Table 3). In T1 compared to T0, a statistically significant increase was observed in the angulation angles of teeth 13 and 16 in the extraction cases, while no significant difference was found in the non-extraction cases. On the other hand, while a statistically significant decrease was observed in the angulation angles of teeth 23 and 26 in the non-extraction cases, no significant difference was found in the extraction cases. The increase in the mesiodistal angulations of teeth 33 and 43 and the decrease in the angulations of teeth 36 and 46 were found to be statistically significant in both the extraction and non-extraction cases (Table 4).

There were no statistically significant differences between T1 and T0 in the angulation angles of teeth 13 and 16 in either females or males. While there was no significant difference between T1 and T0 in the angulations of teeth 23 and 26 in males, a statistically significant decrease was found in T1 compared to T0 in females. In both females and males, the increase in the angulations of teeth 33 and 43 and the decrease in the angulations of teeth 36 and 46 were found to be statistically significant (Table 5). No statistically significant differences were found between the angulations of teeth 13 and 16 measured at T0 and T1 in any skeletal classes. The decrease in the angulations of teeth 23 and 26 in T1 compared to T0 was statistically significant in all skeletal classes. The increase in the angulations of teeth 33 and 43 in T1 compared to T0 was found to be significant in all skeletal classes. While the decrease in the angulations of teeth 36 and 46 in T1 compared to T0 was statistically significant in skeletal class 1 and class 2, no significant difference was found between T0 and T1 in skeletal class 3 (Table 6).

While the increase in the angulation angle of tooth 13 in T1 compared to T0 was significant in angle class 2, it was not statistically significant in angle classes 1 and 3. The decrease in the angulation of tooth 23 was statistically significant in all angle classes. It was found that the angulations of tooth 16 did not show a statistical difference between T0 and T1 in any angle classes. While the decrease in angle class 2 of the angulation of tooth 26 was significant, it was not found to be significant in angle classes 1 and 3. A statistically significant increase was observed in the angulations of teeth 33 and 43 in T1 compared to T0 in all angle classes. While the decreases in angle classes 2 and 3 were found to be significant in the angulation of tooth 36, the decrease in angle class 1 was not significant. However, while the decreases in angle classes 1 and 2 were found to be significant in the angulation of tooth 46, the decrease in angle class 3 was not significant (Table 7). There was no significant difference between the angulations of teeth 13 and 16, measured at T0 and T1, in extraction cases for both miniscrews used and non-used. However, a significant decrease was found in T1 compared to T0 in the angulations of teeth 23 and 26 in both groups. Again, the increase in the angulations of 33 and 43 and the decrease in the angulations of 36 and 46 were found to be statistically significant in T1 compared to T0, in both the miniscrew used and non-used groups (Table 8).

The T0/T1 angulation changes of all the teeth did not show a statistically significant difference between females and males. However, the T0/T1 angulation changes of all the teeth, except teeth 33 and 43, showed significant differences between the extraction and non-extraction cases. Statistically significant differences were found in the T0/T1 angulation changes of all the teeth, except teeth 13, 33, and 43, between the miniscrew used and non-used groups in the extraction cases. No significant difference was found between the skeletal classes, in terms of the T0/T1 angulation angle changes of all the teeth. When the angle classes were compared, no significant difference was found between the angle classes and the changes in the T0/T1 angulation angles of all the teeth, except for tooth 36. While the amount of change in the angulation of tooth 36 was statistically significant, although less significant in angle class 1 compared to classes 2 and 3, no significant difference was found between the amount of change in angle classes 2 and 3. The statistical results are provided in Table 9 and Table 10.

## 4. Discussion

In this study, the aim was to investigate the angular changes in the mesiodistal axial angulation of the canine and first molars after fixed orthodontic treatment. For this, angular measurements were performed on panoramic radiographs, which cause less radiation exposure, are inexpensive, and are commonly used in orthodontics [23].

Although it has been reported that differences of more than 5° cause significant changes in the evaluation of axial angulations with a constructed reference plane in panoramic radiographs, it has been shown in previous studies that variations of 10° or less do not significantly affect tooth angulations [23,27]. In this study, the mesiodistal axial angulations of the teeth were measured to consider the problems inherent in panoramic radiography. However, excellent intraobserver reliability was found between the repeated measurements in randomly selected samples in the study.

Morais et al. [20] examined the changes in the mesiodistal angulations of the maxillary central and lateral incisors after orthodontic treatment on panoramic radiographs in patients with upper anterior diastema. The researchers reported that the decrease in the mesiodistal angulations of the lateral incisors was statistically significant between the pre- and post-treatment, yet not in the central incisors. In our study, when all teeth were compared between T0 and T1, significant differences were found in the mesiodistal axial angulations of all teeth, except in teeth 13 and 16. While there was a statistically significant decrease in the angulation angles of teeth 23, 36, and 46, a statistically significant increase was observed in teeth 33 and 43. Moreover, we emphasize that there was an increase in the angulation angle due to the mesial tipping of the lower canines in all groups and a decrease in the angulation angle due to the distal uprighting in the lower first molars.

Livas et al. [28], in their study, investigated the mesiodistal molar angulation changes in class 2 subdivision cases with unilateral first molar extraction and reported that there was a greater decrease in the angulation angle of the second molar tooth in the extraction cases, compared to the non-extraction cases. They also reported that extraction was an important determinant of angulation, while gender was not. In our study, while there was a significant increase in the angulation angles of teeth 13 and 16 in the extraction cases, no significant difference was found in the non-extraction cases. On the other hand, while there was no significant difference in the angulations of teeth 23 and 26 in the extraction cases, a significant decrease was found in the non-extraction cases. We hypothesize that these differences are due to the extraction of the different teeth in the extraction cases and the differences in the design of the studies. Although the decrease in the angulation angle of teeth 23 and 26 in females was statistically significant, it was observed that the angulation angle changes in females and males were similar.

In another study by Jesuino et al. [29], regarding the mesiodistal angulation angles in the literature, the mesiodistal angulations of the permanent canine and first premolars were investigated. In their study, they found that the angulation angles of the upper canines were significantly higher in males than in females, although they did not find a significant difference between gender in the angulations of other teeth. In our study, we did not find any significant difference in the T0/T1 angulation angle changes between females and males, in any tooth.

Ming et al. [30] reported that they did not find a significant difference between the angulations of the lower and upper first molars before treatment, before orthognathic surgery, and after treatment in patients with skeletal class 3 and facial asymmetry. Similarly, in our study, while there was no significant change in the angulations of the lower and upper first molars in skeletal class 3, between the before and after treatment, mostly significant angulation changes were found in skeletal classes 1 and 2.

You et al. [31] investigated the effect of premolar extraction on the angulation change in second molars. While there was no significant difference between the extraction and control groups in the angulation change, they found a statistically significant difference in the non-extraction group. In the study, T0/T1 angulation changes of all teeth, except teeth 33 and 43, showed significant differences between the extraction and non-extraction groups.

Su et al. [18], in their study investigating the mesiodistal angulations of the first molars, reported that the upper first molar tipped the most distally in angle class 2 and skeletal class 2, while the lower first molar tipped the most mesially, and the opposite was seen in class 3. In the study, while there was a significant decrease in the angulation of teeth 36 and 46 in skeletal classes 1 and 2, the decrease in class 3 was not statistically significant. However, a statistically significant increase was observed in the angulations of teeth 33 and 43, in both the angle and skeletal classes, due to mesial tipping. In addition, while there was no significant difference between the angle and skeletal classes in the T1/T0 angulation changes, statistically significantly fewer changes were found only in angle class 1 compared to classes 2 and 3.

In the literature, there are almost no studies on the effect of orthodontic miniscrew use on mesiodistal axial angulation. In one of them, a study by Vela-Hernández et al. [32], in which the effect of miniscrew use on buccal angulation during incisor intrusion was investigated, a statistically significant increase in buccal angulation was reported in the group using one miniscrew, compared to the group using two miniscrews. This situation might be attributed to the fact that the use of two miniscrews provides more bodily movement and, therefore, less tipping than the use of one miniscrew. In the study, it was observed that there were statistically significant similar differences between T1 and T0 in all teeth, except teeth 23 and 26, in both the groups where the miniscrew was used and not used in the extraction cases. Furthermore, a significant difference was found between the groups, with and without miniscrews, in the T0/T1 angulation angle changes of teeth 23, 16, 26, 36, and 46.

The complexities in the response of the anterior teeth to forces and moments during retraction are difficult to clarify. In this context, Cozzani et al. [33] reported that anterior teeth exhibited varying amounts of uncontrolled tipping in their study, where they compared the effect of different bracket systems on upper anterior retraction. The researchers reported that uncontrolled tipping, which occurs with greater movement of the crown than the root apex in the sagittal plane, is mostly seen in the MBT system, and this is due to the greater play between the bracket slot and the archwire in the MBT system [33,34]. In the study, it is thought that the different angulation changes observed in the upper teeth, in the extraction and miniscrew groups, are due to the uncontrolled tipping movements caused by the MBT bracket system.

In a micro-osteoperforation study by Jaiswal et al. [35], they investigated canine angulation changes on periapical films during retraction using miniscrews in maxillary first premolar extraction cases. Here, they reported that they did not find significant differences between the canine angulation changes. In the study, angulation changes in tooth 23, in the extraction cases, and tooth 13, in the non-extraction cases, were not significant, while a statistically significant increase was found in the angulations of teeth 33 and 43, in both the extraction and non-extraction cases.

The results of the study provide a significant amount of comprehensive data to the literature. However, the limitation of this study is that the accuracy of the angular measurements is relative due to the larger imaging of the craniofacial structures on panoramic radiographs [28]. In addition, the study’s strengths are based upon the advantages of panoramic radiographs, which have been mentioned, rather than cone-beam volumetric tomography, which can provide more precise information, yet is not currently widely used in orthodontics [36].

## 5. Conclusions

It was seen that orthodontic treatment changed the mesiodistal axial angulations of the canine and first molars. While there were no significant changes in the angulation angles of the upper canine and first molars in males between T0 and T1, the decrease in the angulations of teeth 23 and 26 was found to be significant in females. Regardless of gender, tooth extraction, use of miniscrews, angle classification, and skeletal classification, the increase in the angulations of teeth 33 and 43 and the decrease in the angulations of teeth 36 and 46 were notable in all groups.

It was concluded, according to this study, that since the canine and first molar angulations in the maxilla showed differences between the groups, angulation changes should be well controlled in cases where treatment first began in the maxilla. This condition also showed that there is a need for further studies, in which different angulation changes in the maxillary teeth are addressed in a more comprehensive manner. In addition, attention should be paid to the tendency of similar angulation changes that occur in the canine and first molars of the mandible, in all cases.

## Figures and Tables

**Table 1:** Patients’ characteristics included in the study.

Variables	Groups	Nn		%		Total
Gender	Female	237		67.1		100%
	Male	116		32.9		
Treatment	Extraction	158		44.8		100%
	Non-extraction	195		55.2		
Miniscrew	Used	95		60.1		100%
	Non-used	63		39.9		
			Extraction (N(n)		Non-extraction (N(n)	
Skeletal classification	Class 1	159	80	45.1	79	100%
	Class 2	123	59	34.8	64	
	Class 3	71	19	20.1	52	
Angle classification	Class 1	113	40	32	73	100%
	Class 2	179	91	50.7	88	
	Class 3	61	27	17.3	34	

Nn: sample size; %: percentage.

**Table 2:** Angular and linear cephalometric statistics of malocclusions.

		Overjet ^mm^ Mean ± SD	Overbite ^mm^ Mean ± SD	ANS-PNS ^mm^ Mean ± SD	Wits Appraisal ^mm^ Mean ± SD	SN to Go-GN ° Mean ± SD	*Y*-axis ° Mean ± SD	Nasolabial Angle ° Mean ± SD
Skeletal	Class 1	3.78 ± 2.06	2.30 ± 1.87	49.86 ± 5.07	−0.63 ± 3.44	33.10 ± 5.58	61.86 ± 4.27	109.06 ± 11.47
	Class 2	5.30 ± 2.69	3.25 ± 2.39	51.56 ± 5.91	2.33 ± 3.03	34.52 ± 7.31	61.60 ± 4.96	113.26 ± 13.54
	Class 3	1.73 ± 2.83	2.35 ± 2.20	49.24 ± 3.97	−0.96 ± 4.30	34.25 ± 6.14	61.99 ± 4.68	112.51 ± 12.24
Angle	Class 1	3.67 ± 2.48	2.13 ± 1.94	49.65 ± 5.01	−1.22 ± 3.44	33.52 ± 6.14	61.93 ± 4.73	109.42 ± 12.32
	Class 2	4.33 ± 2.67	2.96 ± 2.28	51.05 ± 5.59	1.47 ± 3.45	33.41 ± 6.69	61.32 ± 4.45	111.21 ± 11.69
	Class 3	1.48 ± 2.80	2.68 ± 2.11	49.46 ± 4.37	−0.06 ± 4.24	35.62 ± 5.44	62.99 ± 4.53	114.70 ± 14.47

^mm^: millimeter; °: degree; SD: standard deviation.

**Table 3:** Results of T0/T1 angulation changes for all cases.

	T0	T1	T0/T1 Differences	
Teeth	Mean ± SD	Mean ± SD	Mean ± SD	*p*
**13**	90.84 ± 6.01	91.86 ± 5.76	1.01 ± 7.48	0.055
**23**	91.20 ± 6.07	89.60 ± 5.61	−1.59 ± 7.49	0.000 *
**16**	98.25 ± 6.86	98.75 ± 7.57	0.50 ± 8.17	0.246
**26**	98.47 ± 6.73	97.35 ± 7.26	−1.12 ± 8.05	0.009 *
**33**	90.12 ± 6.21	98.42 ± 8.29	8.29 ± 9.71	0.000 *
**43**	94.77 ± 6.94	99.97 ± 6.48	5.19 ± 8.76	0.000 *
**36**	112.55 ± 6.54	110.38 ± 6.35	−2.17 ± 7.80	0.000 *
**46**	113.73 ± 6.92	111.18 ± 6.70	−2.55 ± 7.53	0.000 *

T0: pretreatment; T1: posttreatment; SD: standard deviation; *p*: significance value; *: *p* < 0.05.

**Table 4:** Results of T0/T1 angulation changes in extraction and non-extraction cases.

	Extraction (n = 158)			Non-Extraction (n = 195)		
Teeth	T0 (Mean ± SD)	T1 (Mean ± SD)	*p*	T0 (Mean ± SD)	T1 (Mean ± SD)	*p*
**13**	90.51 ± 6.17	92.35 ± 5.47	0.001 *	91.26 ± 5.80	91.25 ± 6.07	0.988
**23**	90.80 ± 6.19	90.14 ± 5.06	0.187	91.69 ± 5.90	88.95 ± 6.18	0.000 *
**16**	98.09 ± 7.10	99.98 ± 7.62	0.001 *	98.44 ± 6.56	97.23 ± 7.25	0.064
**26**	98.42 ± 6.89	98.64 ± 7.14	0.695	98.54 ± 6.56	95.75 ± 7.11	0.000 *
**33**	89.88 ± 5.84	98.21 ± 9.04	0.000 *	90.41 ± 6.64	98.68 ± 7.28	0.000 *
**43**	94.91 ± 6.04	100.31 ± 6.43	0.000 *	94.60 ± 7.93	99.55 ± 6.53	0.000 *
**36**	112.05 ± 6.88	110.73 ± 6.07	0.002 *	113.18 ± 6.06	109.95 ± 6.67	0.000 *
**46**	113.03 ± 6.42	111.77 ± 6.03	0.011 *	114.59 ± 7.43	110.45 ± 7.40	0.000 *

T0: pretreatment; T1: posttreatment; SD: standard deviation; *p*: significance value; n: sample size; *: *p* < 0.05.

**Table 5:** Results of T0/T1 angulation changes for gender.

	Male (n = 116)			Female (n = 237)		
Teeth	T0 (Mean ± SD)	T1 (Mean ± SD)	*p*	T0 (Mean ± SD)	T1 (Mean ± SD)	*p*
**13**	89.46 ± 6.37	90.63 ± 5.50	0.120	91.52 ± 5.71	92.46 ± 5.80	0.106
**23**	90.01 ± 6.03	88.95 ± 4.84	0.120	91.78 ± 6.02	89.93 ± 5.94	0.000 *
**16**	96.65 ± 6.13	97.72 ± 6.78	0.098	99.02 ± 7.07	99.25 ± 7.89	0.686
**26**	98.02 ± 6.04	96.79 ± 6.70	0.078	98.70 ± 7.05	97.62 ± 7.52	0.049 *
**33**	91.08 ± 6.30	99.81 ± 6.51	0.000 *	89.65 ± 6.13	97.73 ± 8.97	0.000 *
**43**	95.86 ± 5.78	99.96 ± 6.33	0.000 *	94.24 ± 7.40	99.98 ± 6.56	0.000 *
**36**	111.78 ± 5.81	109.73 ± 6.34	0.002 *	112.93 ± 6.85	110.70 ± 6.34	0.000 *
**46**	111.83 ± 6.66	109.54 ± 6.71	0.002 *	114.66 ± 6.87	111.99 ± 6.56	0.000 *

T0: pretreatment; T1: posttreatment; SD: standard deviation; *p*: significance value; n: sample size; *: *p* < 0.05.

**Table 6:** Results of T0/T1 angulation changes for skeletal classifications.

	Skeletal Class 1 (n = 159)			Skeletal Class 2 (n = 123)			Skeletal Class 3 (n = 71)		
Teeth	T0 (Mean ± SD)	T1 (Mean ± SD)	*p*	T0 (Mean ± SD)	T1 (Mean ± SD)	*p*	T0 (Mean ± SD)	T1 (Mean ± SD)	*p*
**13**	90.77 ± 6.02	91.62 ± 5.60	0.169	90.94 ± 6.19	92.27 ± 5.73	0.108	90.85 ± 5.75	91.68 ± 6.22	0.328
**23**	91.40 ± 6.19	89.80 ± 4.98	0.008 *	91.09 ± 5.24	89.65 ± 6.01	0.023 *	90.92 ± 7.13	89.08 ± 6.24	0.056
**16**	97.72 ± 6.15	98.25 ± 6.56	0.374	98.25 ± 6.94	99.05 ± 7.83	0.297	99.42 ± 8.06	99.34 ± 9.11	0.944
**26**	98.57 ± 6.38	96.84 ± 6.54	0.004 *	98.03 ± 6.49	97.10 ± 7.26	0.214	99.03 ± 7.88	98.93 ± 8.58	0.929
**33**	90.31 ± 5.68	97.56 ± 9.31	0.000 *	89.50 ± 6.95	98.54 ± 7.49	0.000 *	90.78 ± 5.98	100.12 ± 6.90	0.000 *
**43**	95.22 ± 6.63	99.55 ± 6.24	0.000 *	94.31 ± 7.80	100.02 ± 6.62	0.000 *	94.56 ± 6.02	100.82 ± 6.76	0.000 *
**36**	111.84 ± 6.70	109.84 ± 5.93	0.001 *	114.15 ± 6.28	110.92 ± 6.10	0.000 *	111.40 ± 6.18	110.64 ± 7.58	0.447
**46**	112.64 ± 6.21	110.18 ± 5.93	0.000 *	115.33 ± 7.23	111.75 ± 6.68	0.000 *	113.42 ± 7.47	112.42 ± 8.02	0.294

T0: pretreatment; T1: posttreatment; SD: standard deviation; *p*: significance value; n: sample size; *: *p* < 0.05.

**Table 7:** Results of T0/T1 angulation changes for angle classifications.

	Angle Class 1 (n = 113)			Angle Class 2 (n = 179)			Angle Class 3 (n = 61)		
Teeth	T0 (Mean ± SD)	T1 (Mean ± SD)	*p*	T0 (Mean ± SD)	T1 (Mean ± SD)	*p*	T0 (Mean ± SD)	T1 (Mean ± SD)	*p*
**13**	91.38 ± 5.69	91.80 ± 5.45	0.548	90.72 ± 6.53	92.28 ± 5.95	0.008 *	90.22 ± 4.88	90.76 ± 5.71	0.539
**23**	90.99 ± 6.09	89.55 ± 4.98	0.041 *	91.30 ± 5.83	89.83 ± 5.74	0.010 *	91.29 ± 6.79	89.03 ± 6.33	0.024 *
**16**	98.35 ± 6.60	98.71 ± 7.08	0.642	97.52 ± 6.61	98.59 ± 7.25	0.070	100.19 ± 7.71	99.31 ± 9.29	0.454
**26**	98.62 ± 6.70	97.47 ± 6.80	0.100	98.07 ± 5.97	96.58 ± 7.02	0.014 *	99.38 ± 8.65	99.37 ± 8.44	0.991
**33**	89.78 ± 5.89	99.04 ± 7.09	0.000 *	89.89 ± 6.57	97.67 ± 9.40	0.000 *	91.42 ± 5.59	99.46 ± 6.61	0.000 *
**43**	95.88 ± 8.38	100.90 ± 6.27	0.000 *	94.39 ± 6.11	99.32 ± 6.56	0.000 *	93.83 ± 6.08	100.15 ± 6.48	0.000 *
**36**	111.05 ± 7.43	110.62 ± 5.86	0.586	113.05 ± 5.93	110.12 ± 6.28	0.000 *	113.89 ± 6.09	110.70 ± 7.43	0.003 *
**46**	112.85 ± 6.74	111.04 ± 6.08	0.008 *	114.27 ± 6.57	111.11 ± 6.57	0.000 *	113.80 ± 8.13	111.65 ± 8.13	0.064

T0: pretreatment; T1: posttreatment; SD: standard deviation; *p*: significance value; n: sample size; *: *p* < 0.05.

**Table 8:** Results of T0/T1 angulation changes of cases used and non-used miniscrew in extraction cases.

	Miniscrew Non-Used (n = 65)			Miniscrew Used (n = 93)		
Teeth	T0 (Mean ± SD)	T1 (Mean ± SD)	*p*	T0 (Mean ± SD)	T1 (Mean ± SD)	*p*
**13**	91.81 ± 5.05	91.67 ± 6.01	0.853	90.88 ± 6.27	90.96 ± 6.13	0.916
**23**	92.32 ± 5.53	90.22 ± 5.10	0.025 *	91.24 ± 6.14	88.06 ± 6.71	0.000 *
**16**	97.70 ± 6.57	96.34 ± 6.65	0.128	98.95 ± 6.54	97.85 ± 7.62	0.231
**26**	97.31 ± 5.77	95.51 ± 6.40	0.026 *	99.40 ± 6.96	95.92 ± 7.61	0.000 *
**33**	90.50 ± 6.41	99.37 ± 7.83	0.000 *	90.34 ± 6.84	98.19 ± 6.86	0.000 *
**43**	95.09 ± 7.28	101.12 ± 5.75	0.000 *	94.25 ± 8.38	98.46 ± 6.85	0.000 *
**36**	112.64 ± 5.50	110.15 ± 6.92	0.007 *	113.56 ± 6.43	109.81 ± 6.52	0.000 *
**46**	114.39 ± 7.37	110.31 ± 7.32	0.000 *	114.74 ± 7.51	110.55 ± 7.49	0.000 *

T0: pretreatment; T1: posttreatment; SD: standard deviation; *p*: significance value; n: sample size; *: *p* < 0.05.

**Table 9:** Comparison of T0/T1 angulation differences of teeth 13, 23, 16, and 26 between groups for gender, skeletal, and angle classification.

	13		23		16		26	
	T0/T1 Mean ± SD	*p*	T0/T1 Mean ± SD	*p*	T0/T1 Mean ± SD	*p*	T0/T1 Mean ± SD	*p*
**Gender ^+^**								
Males	1.16 ± 8.03	0.789	−1.06 ± 7.31	0.356	0.22 ± 8.73	0.328	−1.07 ± 8.34	0.867
Females	0.94 ± 7.21		−1.85 ± 7.59		1.06 ± 6.90		−1.22 ± 7.44	
**Treatment ^+^**								
Extraction	−0.01 ± 7.14	0.021 *	−2.73 ± 7.95	0.01 *	−1.20 ± 8.13	0.000 *	−2.78 ± 7.97	0.000 *
Non-extraction	1.84 ± 7.66		−0.66 ± 6.99		1.89 ± 7.95		0.22 ± 7.88	
**Miniscrew ^+^**								
Used	0.08 ± 7.74	0.161	−3.22 ± 8.29	0.023 *	−1.02 ± 8.79	0.034 *	−3.46 ± 8.84	0.001 *
Non-used	1.35 ± 7.37		−1.00 ± 7.11		1.05 ± 7.87		−0.27 ± 7.58	
**Skeletal Classification ^++^**								
Class 1	0.85 ± 7.78	0.847	−1.60 ± 7.52	0.937	0.53 ± 7.50	0.768	−1.73 ± 7.48	0.342
Class 2	1.32 ± 7.34		−1.43 ± 7.23		0.80 ± 8.39		−0.92 ± 8.24	
Class 3	0.83 ± 7.09		−1.84 ± 7.96		−0.07 ± 9.25		−0.09 ± 8.89	
**Angle Classification ^++^**								
Class 1	0.41 ± 7.38	0.388	−1.43 ± 7.40	0.746	0.35 ± 8.15	0.269	−1.14 ± 7.32	0.465
Class 2	1.55 ± 7.76		−1.46 ± 7.54		1.06 ± 7.83		−1.49 ± 8.03	
Class 3	0.53 ± 6.77		−2.26 ± 7.61		−0.87 ± 9.09		−0.01 ± 9.32	

^+^: independent sample *t*-test; ^++^: one-way ANOVA test; T0/T1: differences between T0 and T1 measurement; SD: standard deviation; *p*: significance value; *: *p* < 0.05.

**Table 10:** Comparison of T0/T1 angulation differences of teeth 33, 43, 36, and 46 between groups for gender, skeletal, and angle classification.

	33		43		36		46	
	T0/T1 Mean ± SD	*p*	T0/T1 Mean ± SD	*p*	T0/T1 Mean ± SD	*p*	T0/T1 Mean ± SD	*p*
**Gender ^+^**								
Males	8.08 ± 10.22	0.562	4.10 ± 7.35	0.099	−2.04 ± 7.08	0.831	−2.67 ± 7.50	0.654
Females	8.08 ± 10.22		5.73 ± 9.34		−2.23 ± 8.14		−2.67 ± 7.50	
**Treatment ^+^**								
Extraction	8.27 ± 9.61	0.961	4.95 ± 9.75	0.640	−3.23 ± 7.67	0.021 *	−4.14 ± 8.04	0.000 *
Non-extraction	8.32 ± 9.82		5.39 ± 7.89		−1.31 ± 7.82		−1.26 ± 6.85	
**Miniscrew ^+^**								
Used	7.86 ± 9.53	0.614	4.18 ± 10.35	0.192	−3.68 ± 7.93	0.028 *	−4.09 ± 8.64	0.036 *
Non-used	8.45 ± 9.79		5.56 ± 8.10		−1.62 ± 7.69		−1.99 ± 7.02	
**Skeletal Classification ^++^**								
Class 1	7.25 ± 10.23	0.185	4.33 ± 8.19	0.227	−1.99 ± 7.67	0.097	−2.45 ± 7.00	0.071
Class 2	9.04 ± 9.35		5.70 ± 10.01		−3.22 ± 7.59		−3.57 ± 7.84	
Class 3	9.34 ± 9.02		6.25 ± 7.53		−0.75 ± 8.27		−0.99 ± 7.96	
**Angle Classification ^++^**								
Class 1	9.25 ± 8.59	0.438	5.02 ± 10.51	0.549	−0.43 ± 8.41	0.015 *	−1.80 ± 7.06	0.293
Class 2	7.77 ± 10.72		4.93 ± 7.84		−2.92 ± 7.15		−3.16 ± 7.4	
Class 3	8.04 ± 8.51		6.31 ± 7.77		−3.19 ± 8.05		−2.14 ± 8.44	

^+^: independent sample *t*-test; ^++^: one-way ANOVA test; T0/T1: differences between T0 and T1 measurement; SD: standard deviation; *p*: significance value; *: *p* < 0.05.

## Data Availability

All data supporting the results of this study are included within the article. The data is currently not publicly available as it will be used in another still-in-progress study.
